# Genomic Sequence of a Clinical Vancomycin-Resistant Reference Strain, *Enterococcus faecalis* ATCC 51299

**DOI:** 10.1128/genomeA.01495-15

**Published:** 2015-12-17

**Authors:** Kidon Sung, Saeed Khan, Bernard Marasa, Seonggi Min, Ohgew Kweon, Mohamed Nawaz, Carl Cerniglia

**Affiliations:** a National Center for Toxicological Research (NCTR), Division of Microbiology, U.S. Food and Drug Administration (FDA), Jefferson, Arkansas, USA; b Center for Drug Evaluation and Research (CDER), Division of Microbiological Assessment, Office of Process and Facilities, U.S. Food and Drug Administration (FDA), Silver Spring, Maryland, USA; c National Center for Toxicological Research (NCTR), Office of Scientific Coordination, U.S. Food and Drug Administration (FDA), Jefferson, Arkansas, USA

## Abstract

In this paper, we present a draft genome sequence of a quality control reference strain, *Enterococcus faecalis* ATCC 51299 (multilocus sequencing type [MLST] ST6), which is sensitive to teicoplanin but resistant to vancomycin. It is used in an agar screening test for streptomycin, gentamicin, and vancomycin resistance and the resistance marker *vanB*.

## GENOME ANNOUNCEMENT

Vancomycin-resistant enterococci (VRE) (*Enterococcus faecium* and *Enterococcus faecalis*) with high levels of resistance to vancomycin have been reported to harbor the transferable resistance markers *vanA* and *vanB* on the transposable genetic elements Tn*1546* and Tn*5382*, respectively (1, 2). The reference strain used in this study, *E. faecalis* ATCC 51299, was isolated from a peritoneal fluid sample from a patient in St. Louis, MO, USA, and is used in validating antimicrobial susceptibility for vancomycin, streptomycin, and gentamicin (3). It is sensitive to ciprofloxacin, ofloxacin, tetracycline, and bacitracin but resistant to gentamicin, kanamycin, streptomycin, and erythromycin, and it shows interesting vancomycin susceptibility profiles in different growth media (4). The Vitek method and disk diffusion assay indicate a vancomycin-resistant phenotype for *E. faecalis* ATCC 51299 in brain heart infusion agar but a sensitive phenotype on Mueller-Hinton agar plates (4). Automatic susceptibility tests do not always accurately predict the susceptibility of enterococcal isolates (5), and the choice of medium seems to affect the antimicrobial susceptibility profile as well. This might result in the selection of the wrong antimicrobial therapy for life-threatening infections by VRE isolates. The reference strain reported here exhibits the vancomycin resistance gene *vanB* by both PCR and transcriptomic gene expression assays (6). Strains with *vanA*/*vanB* genotypes are responsible for the spread of vancomycin resistance due to their ability to transfer the resistance genes to other organisms (7, 8). For several years, the frequencies of VRE carrying the *vanA* and *vanB* genes have remained between 8 and 15%, but current trends indicate a rising frequency of the *vanB* gene cluster (9). With the increased occurrence of *vanB*-positive VRE, it is important to catalog a genomic database for *E. faecalis* strains that share similar genetic makeup but carry different antimicrobial resistance and/or virulence markers. The genomic sequence data for the reference strain *E. faecalis* ATCC 51299, in comparison with those of other genomic sequences of VRE isolates, will help in understanding the evolutionary process of these isolates and may result in improved mitigation strategies and drug development.

The genomic DNA of *E. faecalis* ATCC 51299 was extracted using a MasterPure Gram-positive DNA purification kit (Epicentre Biotechnologies, Madison, WI, USA). A TruSeq DNA library preparation kit (Illumina, San Diego, CA, USA) and TruSeq paired-end cluster kit were used for DNA preparation and cluster generation for sequencing on a HiSeq system, respectively. The Illumina HiSeq 2500 system was used for sequencing. *De novo* assembly of a total of 30,038,624 high-quality paired-end reads (100 bp in length) was conducted using CLC Genomics Workbench 6.5.1 (CLC bio, Cambridge, MA, USA), and further genome annotation was performed using an annotation method, GeneMarkS+, in the NCBI Prokaryotic Genome Annotation Pipeline (http://www.ncbi.nlm.nih.gov/genomes/static/Pipeline.html). The draft genome sequence of *E. faecalis* ATCC 51299 was 3,232,969 bp in length, with a G+C content of 37.3%, distributed in 70 contigs (*N*_50_ length, 116,948; average coverage, 1,377.0×), with 3,230 coding sequences (CDSs), 3,333 genes, 46 pseudogenes, 19 frameshifted genes, 56 tRNAs, and 1 noncoding RNA (ncRNA).

### Nucleotide sequence accession numbers.

This whole-genome shotgun project has been deposited at DDBJ/EMBL/GenBank under the accession no. JSES00000000. The version described in this paper is version JSES00000000.1.

## References

[1] Arthur M, Molinas C, Depardieu F, Courvalin P 1993 Characterization of Tn*154*6, a Tn*3*-related transposon conferring glycopeptide resistance by synthesis of depsipeptide peptidoglycan precursors in *Enterococcus faecium* BM4147. J Bacteriol 175:117–127. 8380148 10.1128/jb.175.1.117-127.1993 PMC196104

[2] Dahl KH, Rokenes TP, Lundblad EW, Sundsfjord A 2003 Nonconjugative transposition of the *vanB*-containing Tn*5382*-like element in *Enterococcus faecium*. Antimicrob Agents Chemother 47:786–789. doi:10.1128/AAC.47.2.786-789.2003. 12543693 PMC151725

[3] Swenson JM, Clark NC, Sahm DF, Ferraro MJ, Doern G, Hindler J, Jorgensen JH, Pfaller MA, Reller LB, Weinstein MP 1995 Molecular characterization and multilaboratory evaluation of *Enterococcus faecalis* ATCC 51299 for quality control of screening tests for vancomycin and high-level aminoglycoside resistance in enterococci. J Clin Microbiol 33:3019–3021. 8576364 10.1128/jcm.33.11.3019-3021.1995 PMC228625

[4] Nayak R, Khan SA, Watson RH, Cerniglia CE 2002 Influence of growth media on vancomycin resistance of *Enterococcus* isolates and correlation with resistance gene determinants. FEMS Microbiol Lett 214:159–163. doi:10.1111/j.1574-6968.2002.tb11340.x. 12351224

[5] Tenover FC, Tokars J, Swenson J, Paul S, Spitalny K, Jarvis W 1993 Ability of clinical laboratories to detect antimicrobial agent-resistant enterococci. J Clin Microbiol 31:1695–1699. 8349745 10.1128/jcm.31.7.1695-1699.1993 PMC265616

[6] Khan SA, Sung K, Nawaz MS 2011 A transcriptomic expression array, PCR and disk diffusion analysis of antimicrobial resistance genes in multidrug-resistant bacteria. Agric Food Anal Bacteriol 1:123–139.

[7] Prystowsky J, Siddiqui F, Chosay J, Shinabarger DL, Millichap J, Peterson LR, Noskin GA 2001 Resistance to linezolid: characterization of mutations in rRNA and comparison of their occurrences in vancomycin-resistant enterococci. Antimicrob Agents Chemother 45:2154–2156. doi:10.1128/AAC.45.7.2154-2156.2001. 11408243 PMC90620

[8] Klare I, Konstabel C, Badstübner D, Werner G, Witte W 2003 Occurrence and spread of antibiotic resistances in *Enterococcus faecium*. Int J Food Microbiol 88:269–290. doi:10.1016/S0168-1605(03)00190-9. 14597000

[9] Klare I, Witte W, Wendt C, Werner G 2012 Vancomycin-resistant enterococci (VRE). Recent results and trends in development of antibiotic resistance. Bundesgesundheitsblatt Gesundheitsforschung Gesundheitsschutz 55:1387–1400. (In German.) doi:10.1007/s00103-012-1564-6. 23114437

